# Generic and oral quality of life is affected by oral mucosal diseases

**DOI:** 10.1186/1472-6831-12-2

**Published:** 2012-01-07

**Authors:** Li-Jun Liu, Wen Xiao, Qing-Bo He, Wei-Wen Jiang

**Affiliations:** 1 Department of Oral Mucosal Diseases, Shanghai Ninth People's Hospital, Shanghai Jiao Tong University School of Medicine, Shanghai Key Laboratory of Stomatology, Shanghai Research Institute of Stomatology, No. 639 Zhizaoju Road, Shanghai, 200011, China; 2 Shanghai Ninth People's Hospital, Shanghai Jiao Tong University School of Medicine, No. 227 Chong Qing Nan Road, Shanghai 200025, China

## Abstract

**Background:**

The generic and oral health-related quality of life (QoL) has provided opportunity for investigation of the interrelations among generic health, oral health, and related outcomes. The purpose of this study was to identify the generic and oral QoL in the patients with oral mucosal disease (OMD).

**Methods:**

Five hundred and thirty-eight OMDs were recruited in this study. The instruments applied were Chinese version of the 36-item short form health survey (SF-36) and the short-form of Oral Health Impact Profile (OHIP-14).

**Results:**

The mean score of sum OHIP-14 was significantly higher in the patients with OMD (10.81 ± 9.01) compared with those in the healthy subjects (HS) (6.55 ± 6.73) (p < 0.001, Mann-Whitney U test). 56.51% of the OMD patients and 12.94% of the HS reported at least one oral negative impact (p < 0.001, Chi-square test). The overall mean score of SF-36 was significantly lower in the patients with OMD (74.54 ± 12.77) compared with those in the HS (77.97 ± 12.39) (p = 0.021, t-test).

**Conclusions:**

Administration of specific and generic questionnaires of QoL can provide us a detailed picture of the impact of OMDs on patients, and both generic and oral QoL were impaired in the patients with OMD.

## Background

Oral mucosal diseases (OMDs) are common, and many of them are unknown cause. For example, recurrent aphthous stomatitis (RAS) affects about 0.5-60% of the population [1,2]. There is no medication gives completely reliable relief. Patients with OMD such as pemphigus, which is a rare but serious and highly disabling immunobullous disease of the skin and mucous membranes, can be suffered from life-threatening symptom and be influenced daily life in many ways. Hence, the consequences of OMDs are not only physical, they are also social and psychological. These diseases seriously impair quality of life (QoL) in a large number of individuals and can affect various aspects of life, including oral function, appearance, and interpersonal relationships [3-5]. Information regarding the impact of OMD on QoL is a recognized need. The importance of embracing patients' views in assessing oral health needs and in treatment planning has been advocated. Therefore a number of different patient centered oral health status measures have been developed over the past decade to assess the physical, social and psychological consequences of oral health and the impact of oral health status on QoL. These measures are thought to complement traditional clinical oral health status measures, to improve communication between patients and clinical physicians, and provide greater understanding of the consequences of oral disease upon day to day living and life quality [6,7].

The 36-item short form health survey (SF-36) is designed as a generic indicator of health status with a wide range of types and severity of conditions [8]. The oral health impact profile (OHIP) is an instrument designed to measure oral-health-related QoL. The short-form of OHIP (OHIP-14) is reported to be a useful instrument for use in a clinical setting with good reliability, validity and precision [9].

In the recent study, active oral ulcers were observed to be a significant factor for poor oral health [10]. López-Jornet et al. addressed patients with burning mouth syndrome (BMS) yield poorer scores on all scales of SF-36 and OHIP-49 [11]. The purpose of this study was to evaluate the generic and oral QoL in the patients with OMD using SF-36 questionaire and OHIP-14.

## Methods

### Subjects

This was an observational study to evaluate the self-perceived health-related QoL in the patients with OMD. Five hundred and twenty four first-visting patients diagnosed as various OMDs at the Department of Oral Mucosal Diseases, Shanghai Ninth People's Hospital, Shanghai Jiao Tong University School of Medicine during August of 2009 to April of 2010, were enrolled. Among 524 patients, 14 patients were involved in 2 disorders. Overall, 538 cases of OMD included RAS, oral lichen planus (OLP), burning mouth syndrome (BMS), paraesthesia, candidosis, cheilitis, oral leukoplakia, discoid lupus erythematosus, atrophic glossitis, stomatitis, herpes zoster, geographic tongue, hyperkeratosis, herpes simplex, pemphigus, dry mouth and the other OMDs. We categorized these OMDs into 5 groups: RAS, OLP, BMS & paraesthesia, candidosis, and others, which including the various types of OMDs whose number less than 30. The healthy subjects (HS) were 85 healthy volunteers over 14 years of age. Among them, 29 were recruited from a local community after oral screening by a dentist (L-J. L), and the other 56 were recruited from the family members of patients with OMD studied. The study was approved by the ethics committee of Shanghai Ninth People's Hospital (#200703). The enrolled subjects were given detailed information about the study. The interview was carried out after receiving informed consent from the subjects. The information collected included age, sex, city of origin, and clinical diagnosis. Subjects were given self-administered questionnaires of Chinese version SF-36 and OHIP-14 [12,13].

### Inclusion and exclusion criteria

Inclusion criteria for patients with OMD in the study were: first-visting outpatients at the Department of Oral Mucosal Diseases during the study period, aged over 14 years, and diagnosed as OMD in accordance with clinical findings and/or biopsy. Exclusion criteria for patients with OMD were: 14 or under 14 years or consecutive OMD patients. Inclusion criteria for HS were: After oral screening, volunteers aged over 14 years without OMD, were included. Exclusion criteria for HS were: 14 or under 14 years or OMD patients.

### Questionnaires

The standard version of the Health Questionnaire SF-36 contained 8 areas. Physical functioning, Role limitations physical, Bodily pain, General medical health, Vitality, Social functioning, Role limitations emotional and Mental health. It evaluated the QoL of the people of which 14 years of age and older during the 4 weeks prior to the interview. The higher scores indicated better health; thus, 0 was the worst state of health and 100 the ideal state of health. The Chinese vesion of SF-36 questionnaire used in this study was translated and validated by Li et al [12]. The OHIP-14 contained 7 different domains: Functional limitation items, Physical pain items, Psychological discomfort items, Physical disability items, Psychological disability items, Social disability items and Handicap items. We used Chinese version developed by Xin et al [13]. The OHIP-14 scores were calculated in two ways [14]. The first method was to sum the numeric response codes for all 14 items (sum OHIP-14). For each of the 14 OHIP questions, subjects were asked how frequently they had experienced impact in the preceding 12 months using a 5-point scale coded 4 = very often, 3 = fairly often, 2 = occasionally, 1 = hardly ever and 0 = never. The higher scores indicated the worse oral health. This method was irrespective of their frequency and incorporated the full range of impact responses. The second method was a simple counting (OHIP-14 sc) of the number of items to which a subject responded 'fairly often' or 'very often'. This reduced the response scale to a dichotomy and indicated the frequency of the occurrence of negative impact on a yearly level.

### Statistical analysis

Data was analyzed using the SAS 8.2 statistics program. A descriptive study was made of each variable. Mann-Whitney U test was used to compare the sum OHIP-14 in each group. Independent t-test was used in comparisons of the scores of the groups in SF-36 and age. Chi-square test was used to compare the constituent ratio about fairly often and very often of people reporting social impact items and gender in each group. Probability for p ≤ 0.05 was accepted as significant.

## Results

### Baseline characteristics of patients with OMD and HS

Among the various OMDs (Table 1), patients with RAS contained 76 males (46.63%) with a mean age of 48.5 (SD = 16.2), patients with OLP contained 33 males (27.27%) with a mean age of 49.3 (SD = 16.2), patients with BMS & paraesthesia contained 10 males (23.26%) with a mean age of 51.9 (SD = 15.7), patients with candidosis contained 15 males (40.54%) with a mean age of 50.0 (SD = 17.1), patients with others OMD contained 78 males (44.83%) with a mean age of 47.1 (SD = 17.6) and HS contained 31 males (36.47%) with a mean age of 46.3 (SD = 16.7). No significance of age and gender was found among each group.

**Table 1:** Baseline characteristics of patients with OMD and HS

		Age		Gender	
		
		Years (range)	p	Male (%)	p
Healthy Subjects	n = 85	46.34 ± 16.69 (24-86)		31 (36.47%)	

	RAU (n = 163)	48.52 ± 16.21 (14-80)	0.322	76 (46.63%)	0.125
	OLP (n = 121)	49.32 ± 16.18 (19-83)	0.200	33 (27.27%)	0.160
Oral Mucosal Disease	BMS & Paraesthesia (n = 43)	51.93 ± 15.65 (20-79)	0.070	10 (23.23%)	0.130
	Candidosis (n = 37)	49.62 ± 17.12 (20-78)	0.324	15 (40.54%)	0.670
	Others (n = 174)	47.13 ± 17.64 (14-87)	0.731	78 (44.83%)	0.201
	n = 538	48.60 ± 16.69 (14-87)	0.247	212 (39.41%)	0.606

### The measurement of oral-health-related quality of life in the patients with OMD

OHIP scores were calculated using the summary and simple-count scores method. The distribution of sum OHIP-14 ranged from 0 to 53 was highly skewed; however, the total mean score of sum OHIP-14 was significantly worse in the patients with OMD (10.81 ± 9.01) compared with those in the HS (6.55 ± 6.73, p < 0.001, Mann-Whitney U test). Age (p = 0.247, t-test) and gender (p = 0.606, Chi-square test) did not affect the sum OHIP-14 scores. It indicated that OHIP-14 could discriminate between OMD and HS groups. The mean scores of 2 different domains of OHIP-14, which including Physical pain and Psychological discomfort were significantly worse in the patients with OMD than those in the HS (Table 2). The other 5 domains of OHIP-14, which including Functional limitation, Physical disability, Social disability, Psychological disability and Handicap, showed no difference between OMD and HS. In addition, the Painful aching and Uncomfortable to eat were the two most highly scored items of OHIP-14 in the patients with OMD.

**Table 2:** The sum OHIP-14 in oral mucosal disease and healthy subjects.

	Oral Mucosal Disease		Healthy Subjects		
		
Social Impact Item	mean ± SD	Median (IQR)	mean ± SD	Median (IQR)	p
Functional limitation items	1.40 ± 1.82	1 (0-3)	0.95 ± 1.21	0 (0-2)	0.150
*Trouble pronouncing*	0.49 ± 0.98	0 (0-0)	0.44 ± 0.81	0 (0-1)	0.911
*Taste worsened*	0.91 ± 1.26	0 (0-2)	0.52 ± 0.72	0 (0-1)	0.113
Physical pain items	3.68 ± 2.43	4 (2-6)	1.76 ± 1.68	2 (0-3)	< 0.001
*Painful aching*	1.92 ± 1.32	2 (1-3)	0.94 ± 0.94	1 (0-2)	< 0.001
*Uncomfortable to eat*	1.76 ± 1.38	2 (0-3)	0.82 ± 0.92	1 (0-1.5)	< 0.001
Psychological discomfort items	1.48 ± 1.87	1 (0-3)	0.67 ± 1.27	0 (0-1)	< 0.001
*Self-conscious*	0.62 ± 1.05	0 (0-1)	0.34 ± 0.70	0 (0-0.5)	0.077
*Tense*	0.85 ± 1.11	0 (0-2)	0.33 ± 0.73	0 (0-0)	< 0.001
Physical disability items	1.13 ± 1.66	0 (0-2)	0.76 ± 1.10	0 (0-1.5)	0.213
*Diet unsatisfactory*	0.60 ± 1.02	0 (0-1)	0.39 ± 0.66	0 (0-1)	0.319
*Interrupt meals*	0.53 ± 0.93	0 (0-1)	0.38 ± 0.67	0 (0-1)	0.396
Psychological disability items	1.28 ± 1.67	0 (0-2)	1.06 ± 1.42	0 (0-1)	0.393
*Difficult to relax*	0.81 ± 1.11	0 (0-2)	0.67 ± 0.89	0 (0-1)	0.516
*Embarrassed*	0.46 ± 0.84	0 (0-1)	0.39 ± 0.69	0 (0-1)	0.776
Social disability items	1.04 ± 1.45	0 (0-2)	0.74 ± 1.16	0 (0-1)	0.118
*Irritable*	0.73 ± 0.99	0 (0-1)	0.53 ± 0.78	0 (0-1)	0.137
*Difficulty doing jobs*	0.31 ± 0.71	0 (0-0)	0.21 ± 0.54	0 (0-0)	0.308
Handicap items	0.80 ± 1.39	0 (0-1)	0.60 ± 1.09	0 (0-1)	0.271
*Life less satisfying*	0.59 ± 0.96	0 (0-1)	0.44 ± 0.73	0 (0-1)	0.322
*Unable to function*	0.22 ± 0.62	0 (0-0)	0.16 ± 0.51	0 (0-0)	0.584

Total	10.81 ± 9.01	9 (4-15)	6.55 ± 6.73	4 (0.5-10)	< 0.001

SD: standard deviation

IQR: interquartile range

Next, we compared the total mean score of sum OHIP-14 in each group of OMDs with HS. The mean score of sum OHIP-14 was significantly worse in the patients with RAS (16.14 ± 10.05, p < 0.001, Mann-Whitney U test), OLP (8.89 ± 8.02, p = 0.008, Mann-Whitney U test), BMS & paraesthesia (8.88 ± 7.07, p = 0.033, Mann-Whitney U test) and others (8.13 ± 7.19, p = 0.05, Mann-Whitney U test) compared with that in HS (6.55 ± 6.73). But no significant difference was found between the 'Candidosis' group and HS.

Furthermore, to evaluate the frequency of the occurrence of negative impact in the patients with OMD, we catogorized the subjects with responses 'very often' and 'fairly often' in OHIP-14 statement as a negative impact group and analyzed the number of negative impacts. 56.51% of the OMD patients and 12.94% of HS reported at least one oral negative impact over the last year. For all 7 domains of OHIP-14, the OMD patients with significant higher numbers reporting 'very often' and 'fairly often' in comparison with HS (Table 3). There was no negative impact reporting of Diet unsatisfactory, Interrupt meals, Difficulty doing jobs, and Life less satisfying in HS. The lower percentage of negative impact reporting in OMD was the sub-iterm of Embarrassed (2.97%), Difficulty doing jobs (2.60%) and Unable to function (2.04%) (Table 3).

**Table 3:** Number of subjects reporting negative social Impact by OHIP-14 sc.

	Oral Mucosal Disease	Healthy Subjects	
		
Social Impact Item	number (%)	number (%)	p
Functional limitation items	120/538(22.3)	3/85 (3.53)	< 0.001
*Trouble pronouncing*	46/538 (8.55)	2/85 (2.35)	0.047
*Taste worsened*	95/538 (17.66)	1/85 (1.18)	< 0.001
Physical pain items	257/538(47.77)	7/85(8.24)	< 0.001
*Painful aching*	214/538(39.78)	3/85(3.53)	< 0.001
*Uncomfortable to eat*	199/538(36.99)	4/85(4.71)	< 0.001
Psychological discomfort items	90/538(16.73)	4/85 (4.71)	0.004
*Self-conscious*	47/538(8.74)	3/85 (3.53)	0.101
*Tense*	64/538(11.90)	2/85(2.35)	0.008
Physical disability items	60/538(11.15)	0/85(0.00)	0.001
*Diet unsatisfactory*	44/538 (8.18)	0/85(0.00)	0.006
*Interrupt meals*	27/538 (5.02)	0/85(0.00)	0.039^a^
Psychological disability items	62/538(11.52)	3/85(3.53)	0.025
*Difficult to relax*	56/538 (10.41)	2/85 (2.35)	0.018
*Embarrassed*	16/538 (2.97)	1/85(1.18)	0.492^a^
Social disability items	44/538(8.18)	1/85(1.18)	0.021
*Irritable*	37/538(6.88)	1/85(1.18)	0.047^a^
*Difficulty doing jobs*	14/538(2.60)	0/85 (0.00)	0.236^a^
Handicap items	37/538(6.88)	1/85(1.18)	0.041
*Life less satisfying*	36/538(6.69)	0/85(0.00)	0.010^a^
*Unable to function*	11/538 (2.04)	1/85(1.18)	1.000^a^

a. Fisher's exact test: All other p-values refer to Chi-square test.

### The generic quality of life in the patients with OMD

The SF-36 subscale scores did not show significant difference bewteen OMD and HS (p > 0.05), except in the scores of Physical functioning (p = 0.001), Bodily pain (p < 0.001) and General Health (p < 0.001) (Table 4). The overall mean score of SF-36 was significantly lower in the patients with OMD (74.54 ± 12.77) compared with that in HS (77.97 ± 12.39). Age (p = 0.247, t-test) and gender (p = 0.606, Chi-square test) did not affect the SF-36 scores.

**Table 4:** Comparison by SF-36 sub-scale scores in oral mucosal disease and Healthy subjects.

Dimensions	Oral Mucosal Disease	Healthy Subjects	p
Physical functioning	96.65 ± 5.88	92.06 ± 12.52	0.001
Role physical	79.41 ± 33.08	83.82 ± 32.89	0.254
Bodily pain	70.89 ± 23.21	79.77 ± 19.70	< 0.001
General health	58.75 ± 26.17	69.45 ± 19.96	< 0.001
Vitality	72.39 ± 16.56	75.65 ± 14.94	0.089
Social functioning	88.41 ± 16.60	89.02 ± 14.60	0.751
Role emotional	74.16 ± 36.04	81.18 ± 31.05	0.090
Mental health	72.29 ± 15.89	74.49 ± 13.65	0.227

Total	74.54 ± 12.77	77.97 ± 12.39	0.021

Furthermore, we compared the mean score of SF-36 in each group of OMD to HS. The mean score of SF-36 was significantly lower in the patients with RAS (72.66 ± 11.68) (p = 0.001, t-test) and BMS & paraesthesia (69.68 ± 12.64) (p < 0.001, t-test) compared with that in HS. The mean scores of SF-36 did not show significant difference bewteen the other 3 groups with OMD and HS.

## Discussion

The effects of illness on QoL can be related to the impairment, disability and handicap [15]. In this study, we evaluated of generic and oral QoL in overall OMDs during routine clinical activities, and compared the data of OHIP-14 and SF-36 between OMD and HS. The results yield additional information that may be relevant and useful for the clinical management of patients with OMD.

Our study is institutional based research. All patients studied were recruited at the Department of Oral Mucosal Diseases, Shanghai Ninth People's Hospital. Part of the HS were recruited from a local community in Shanghai, which was a relatively stable community. The others were recruited from the family members of patients with OMD studied. Some of the family members of patients refused to participate the study. Therefore the size of HS was lower than that of overall OMD patients.

OMD are common, and many of them are unknown cause. Patients with OMD can be suffered from life-threatening symptom and be influenced daily life in many ways which including a psycho-social effect as well as a functional impact. Measurement of QoL may help to assess unknown cause conditions. Mumcu et al. used OHIP-14 and SF-36 to measure the oral and general health related QoL in the patients with Behçet's disease, RAS and healthy controls, and observed worse oral QoL in these patients [10]. McGrath C et al. evaluated the sensitivity of two patient-centred outcome measures to the topical application of a corticosteroid (betamethasone) in the treatment of OLP by UK Oral Health Related Quality Of Life measure (OHQOL-UK) and OHIP-14 [7]. Previous research results also indicated that clinical oral disease such as BMS and dry mouth could affect life quality using OHIP-14, OHIP-49 and SF-36 [5,11]. Tabolli et al. used both specific and generic instruments including OHIP-14, SF-12, and 12-item General Health Questionnaire questionaires (GHQ-12) to study QoL affected by various oral mucosal conditions [16]. They found that OMDs radically affected QoL and were accompanied by a high frequency of psychological problems. The similar result could be found in the study by Llewellyn et al [17]. They used OHIP-14 questionaire and observed the greatest impairment to QoL was register on physical pain. The measures employed in this study were a oral health-related quality of life instrument, the OHIP-14, and a generic health-related quality of life instrument, the SF-36, which had been widely used internationally. To the best of our knowledge, it is the first time that both of OHIP-14 and SF-36 were used to evaluate QoL in overall OMDs and control HS. We showed that there was a significant lower scores in generic and oral QoL for patients with OMD than that with HS. Our study suggested that the evaluation of the effects of OMD on generic and oral QoL might be considered as a part of clinical decision processes.

Discriminant validity is the validity obtained when we measure two things that are thought to be dissimilar and our measures can discriminate between them. Allen & Locker has previously discussed the discriminant validity of OHIP [14]. They addressed that that the OHIP could discriminate between clinically disparate groups, while the SF-36 did not [18]. Hunt et al. suggested that the SF-36 had an advantage over other similar instruments, such as the Nottingham health profile [19]. Allen et al. reported that generic health can affect a patient's ability to tolerate dentures [18]. Our findings indicated that the SF-36 score could discriminate between overall OMDs and HS as well as sum OHIP-14 did. The probable reason for this was that many OMDs were unknown cause and multisystem involved. However, we noticed that when OMDs categorizing into 5 groups, the mean score of sum OHIP-14 for RAS, OLP, BMS & paraesthesia and others was significantly different from HS. On the other hand, only RAS and BMS & paraesthesia could be discriminated from HS by the mean score of SF-36. The results of the study by Lopez-Jornet P et al. showed that BMS yielded poorer quality of life scores than the control group in all the domains of the questionnaires including OHIP-49 and SF-36 [11]. This study could also show the same result between BMS & paraesthesia and HS. It would be advisable to use these in conjunction with classical instruments for clinical diagnosis [20], meanwhile further verification with large cohort is needed.

While this study showed an overall lower SF-36 score for OMD patients, the result for the Physical Functioning sub-scale showed OMD were actually healthier on this dimension. The probable reasons for this result were: 1, OMDs were mainly confined in the oral cavity. Therefore the influence by OMDs on Physical Functioning was limited. 2, With increased age the influence of Physical Functioning could be influenced by systemic disease. The mean age of the overall respondents was over 45. A limitation of the study was that we did not perform screening for systemic disease. Therefore, 'HS' only means participants without OMD, and does not necessarily mean those without systemic disease.

In this study, the size of overall OMD and HS was not homogeneous. We failed to collect more HS, because some OMD patients' family member rejected to be involved. We also further grouped the observed OMDs into 5 categories. However, we are aware that some grouping may be arbitrary. The group named 'others' included very different OMDs and was created because of small numbers (< 30).

## Conclusions

Administration of specific and generic questionnaires of QoL can provide a detailed picture of the impact of OMDs on patients. Both oral and generic QoL were impaired in the patients with OMD.

## Competing interests

The authors declare that they have no competing interests.

## Authors' contributions

LJL carried out the interviews, participated the data analysis and drafted the manuscript. WX assisted the data analysis. QBH performed the statistical analysis. WWJ conceived of the study, and participated in its design and coordination and helped to draft the manuscript. All authors read and approved the final manuscript.

## Pre-publication history

The pre-publication history for this paper can be accessed here:

http://www.biomedcentral.com/1472-6831/12/2/prepub
